# Circulating Blood-Based Biomarkers in Pulmonary Hypertension

**DOI:** 10.3390/jcm11020383

**Published:** 2022-01-13

**Authors:** Marta Banaszkiewicz, Aleksandra Gąsecka, Szymon Darocha, Michał Florczyk, Arkadiusz Pietrasik, Piotr Kędzierski, Michał Piłka, Adam Torbicki, Marcin Kurzyna

**Affiliations:** 1 Department of Pulmonary Circulation, Thromboembolic Diseases and Cardiology, Centre of Postgraduate Medical Education, European Health Centre Otwock, 05-400 Warsaw, Poland; szymon.darocha@ecz-otwock.pl (S.D.); michal.florczyk@ecz-otwock.pl (M.F.); piotr.kedzierski@ecz-otwock.pl (P.K.); michal.pilka@ecz-otwock.pl (M.P.); adam.torbicki@ecz-otwock.pl (A.T.); marcin.kurzyna@ecz-otwock.pl (M.K.); 2 1st Chair and Department of Cardiology, Medical University of Warsaw, 02-097 Warsaw, Poland; gaseckaa@gmail.com (A.G.); apietrasik@tlen.pl (A.P.)

**Keywords:** pulmonary hypertension, chronic thromboembolic pulmonary hypertension, pulmonary arterial hypertension, biomarkers, right heart failure

## Abstract

Pulmonary hypertension (PH) is a serious hemodynamic condition, characterized by increased pulmonary vascular resistance (PVR), leading to right heart failure (HF) and death when not properly treated. The prognosis of PH depends on etiology, hemodynamic and biochemical parameters, as well as on response to specific treatment. Biomarkers appear to be useful noninvasive tools, providing information about the disease severity, treatment response, and prognosis. However, given the complexity of PH, it is impossible for a single biomarker to be adequate for the broad assessment of patients with different types of PH. The search for novel emerging biomarkers is still ongoing, resulting in a few potential biomarkers mirroring numerous pathophysiological courses. In this review, markers related to HF, myocardial remodeling, inflammation, hypoxia and tissue damage, and endothelial and pulmonary smooth muscle cell dysfunction are discussed in terms of diagnosis and prognosis. Extracellular vesicles and other markers with complex backgrounds are also reviewed. In conclusion, although many promising biomarkers have been identified and studied in recent years, there are still insufficient data on the application of multimarker strategies for monitoring and risk stratification in PH patients.

## 1. Introduction

Pulmonary hypertension (PH) is a progressive, heterogenous disease, characterized by increased pulmonary vascular resistance (PVR), subsequently leading to elevated pulmonary arterial pressure (PAP) and increased workload of the right ventricle (RV). The RV adapts to the pathological afterload by increasing wall thickness and contractility. However, the compensatory mechanisms may fail, resulting in right heart failure (HF) and death, if not properly treated. 

**Figure 1:**
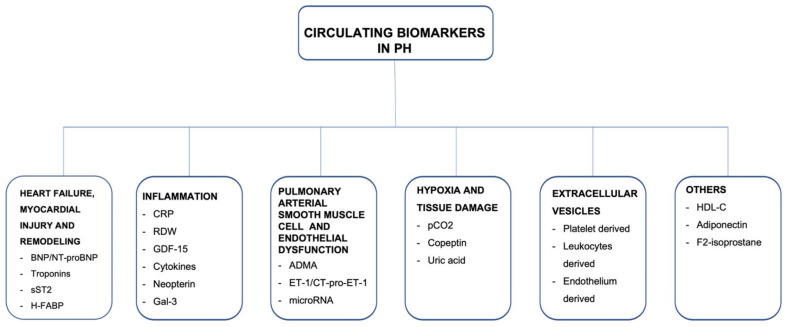
A summary of circulating biomarkers in precapillary pulmonary hypertension.

Consistent with the European Society of Cardiology (ESC)/European Respiratory Society (ERS) Guidelines, there are five groups of PH, according to clinical and pathophysiological criteria. Group 1 contains idiopathic pulmonary arterial hypertension (iPAH), as well as drug-induced PAH and all heritable forms of PAH. Group 2 is PH secondary to left-sided heart failure. PH in group 3 is caused by lung disease and/or hypoxia, and group 4 is chronic thromboembolic pulmonary hypertension (CTEPH). Group 5 consists of PH due to uncertain multifactorial mechanisms. Targeted pharmacological or interventional treatment can be offered to patients diagnosed with PAH and CTEPH, respectively [1,2]. The prognosis of PH varies broadly and depends mostly on etiology of PH, but is also based on hemodynamic and biochemical parameters, which indicate the severity of right ventricular failure, as well as on response to specific treatment. Early recognition of the disease and risk stratification seem to be crucial to identifying patients at high risk and optimizing therapeutic management. Thus, biomarkers may specifically indicate the disease and provide information about the disease stage and treatment response in a relatively easily accessible and noninvasive way. The search for novel emerging biomarkers is still ongoing, resulting in a few potential biomarkers mirroring numerous pathophysiological courses. The main focus is on detecting and quantifying abnormal adaptations and remodeling of the right heart in response to chronic pulmonary circulation impairment. However, in the natural course of PH and right ventricular HF, tissue damage, fibrosis, inflammation, and endothelial dysfunction seem to be also crucial underlying mechanisms, which may be included in noninvasive biomarker assessment (Figure 1). In the present article, we review circulating biomarkers related to different mechanisms underlying the precapillary PH and describe the potential application for them, highlighting their limitations and necessity for further investigation. 

## 2. Biomarkers Related to Heart Failure, Myocardial Injury, and Remodeling

In PH, elevated PVR and PAP lead to hemodynamic stress, myocardial strain, and stretching of the heart. Consequently, this condition results in the release of the molecular mediators, indicative for numerous cardiovascular diseases with additional prognostic value. Several markers associated with HF, myocardial injury, and myocardial remodeling, such as natriuretic peptides, cardiac troponins, soluble ST2, and heart-type fatty acid-binding protein, have been investigated in a cohort of patients with precapillary PH. 

### 2.1. Natriuretic Peptides

Brain-type natriuretic peptide (BNP) is produced as an inactive precursor (proBNP), then converted into the active form N-terminal-pro brain-type natriuretic peptide (NT-proBNP) and released from cardiomyocytes. Due to the longer half-life of NT-proBNP compared to BNP, NT-proBNP is preferred in clinical practice as a marker of heart overload and myocardial dysfunction [3,4]. NT-proBNP remains a well-established and widely used biomarker in numerous cardiovascular diseases. It is released in response to ventricular wall stress and myocardial hypoxia or ischemia. NT-proBNP is mostly used in the diagnostic process of patients with acute or chronic HF as well as in predicting prognosis of those patients [5]. In PH patients, serum NT-proBNP levels correlate with right heart dysfunction and provide prognostic information at diagnosis and during follow-up assessment [6,7,8]. However, due to the high variability of NT-proBNP levels and its possible inadequate correlation with hemodynamic parameters and exercise capacity, it should only be interpreted in the clinical context [6]. At present, NT-proBNP is a crucial element of risk stratification in PAH patients and is addressed in both the risk score developed from the REVEAL registry (Registry to Evaluate Early and Long-Term PAH Disease Management) [9,10] and in the risk stratification method proposed by ESC/ERS guidelines [2]. Consistent with REVEAL registry data, a baseline NT-proBNP level of ≤340 ng/L is a strong predictor of improved survival up to 5 years in PAH patients [10]. In slightly different terms, the ESC/ERS guidelines classify NT-proBNP concentrations as low (<5%), intermediate (5–10%), or high (>10%) risk of 1-year mortality, by using specific thresholds of 300 and 1400 ng/L [2]. A significant decrease in NT-proBNP levels among patients with PAH is associated with the response to targeted medical therapy [11,12]. In CTEPH patients, BNP may not only reflect the degree of RV dysfunction and hemodynamic severity of the disease, but also facilitate to assess the effect of pulmonary endarterectomy (PEA) [13], with estimated BNP baseline cut-off values predictive of worse postoperative survival [14]. Furthermore, both balloon pulmonary angioplasty (BPA) as well as pharmacological treatment result in a decrease of NT-proBNP concentration [15,16,17]. In patients treated with BPA, a reduction in NT-proBNP concentration is associated with a significant decrease in mean PAP and PVR, thereby indicating the procedural success of BPA [15]. Table 1 and Table 2 presents changes in BNP and NT-proBNP concentrations before and after BPA treatment in the hitherto published case series.

### 2.2. Cardiac Troponins

So far, both cardiac troponin I (cTnI) and T (cTnT) are the principal biomarkers for the detection of myocardial damage and key factors in the diagnosis of acute myocardial infarction [29]. In addition, the development of high-sensitivity assays has made it possible to detect troponin concentrations and their association with morbidity and mortality in many chronic diseases, such as heart failure, coronary artery disease, or chronic kidney disease [30,31,32,33]. Although the underlying mechanisms for troponin release in some conditions remain not completely elucidated, in most cases troponins levels correlate with markers of left heart structural abnormalities and other markers related to left HF. In contrast, the mechanism of troponin release in PH patients seems to be associated with RV pathology, seemingly caused by demand–perfusion mismatch or microcirculation impairment. These theories are supported by the results from several research studies, in which significant correlations between troponins concentration and hemodynamic parameters, including mean PAP (mPAP), mixed venous oxygen saturation (mvSatO2), and RV ejection fraction, were identified [34,35]. Moreover, both cTnT and cTnI concentrations were associated with worse outcomes in mixed cohorts of PH patients [34,36]. Thereby, ESC guidelines indicate that for comprehensive prognostic assessment and risk stratification, troponin levels should be measured at the diagnosis of PAH, then at least once a year or whenever the patient presents with clinical worsening [2]. In CTEPH patients undergoing interventional treatment with BPA, high-sensitivity cTnT concentration decreases stepwise under therapy, signifying a reduction of ongoing myocardial damage due to decreased right ventricular afterload after BPA therapy [37]. Thus, also in the CTEPH patient population, troponins can be a useful marker to monitor the progress of treatment.

### 2.3. Soluble ST2

Soluble ST2 (sST2) protein is another promising biomarker in PH patients. Protein ST2 belongs to the Toll interleukin 1 receptor superfamily and exists in two isoforms: transmembrane ST2 ligand (ST2L) and soluble ST2 (sST2), which circulates in the blood. The transmembrane form is expressed mainly on inflammatory cells and takes part in strengthening of the immune response of Th2 lymphocytes. However, it is also exposed in cardiomyocytes and endothelium [38]. The ligand for ST2 is interleukin 33 (IL-33), whose expression increases due to mechanical overload and ischemia of cardiomyocytes [38]. The paracrine IL-33/ST2L system plays a protective role, counteracting fibrosis and myocardial hypertrophy. The sST2 protein, which prevents IL-33 binding to the ST2L, is responsible for interrupting this protective action. The balance between both isoforms of ST2 protein ensures the correct biological effect [38,39]. The increase of sST2 concentration in plasma is associated with cardiac remodeling and hemodynamic stress [38,39,40]. Besides natriuretic peptide family and cardiac troponins, sST2 protein may be an additional biomarker for adverse outcomes in cohorts of patients with acute and chronic heart failure [41,42,43]. The sST2 level above 35 ng/mL in patients with HF is associated with higher risk of adverse events, defined as hospitalization or death in one year, in comparison to subjects with sST2 level below this value [44,45,46]. At present, there is increasing evidence of the use of the sST2 protein for risk stratification in patients with RV failure due to PH. In different types of PH, higher sST2 levels are linked to the remodeling of the RV [47]. In a study involving 100 patients diagnosed with PAH or CTEPH, significant correlations between sST2 and cardiac index (CI), mean right atrial pressure (mRAP), PVR, mvSatO2, NT-proBNP concentration, and 6 min walking distance (6MWD) were noticed [48]. These observations are consistent with those from other studies conducted in smaller populations of patients with precapillary PH [49,50]. Moreover, sST2 has been assessed as a marker of therapy response in 57 CTEPH patients, treated with BPA. In detail, the median sST2 concentration decreased to the range of control group after interventional treatment, but it was not related to the individual grade of response to BPA therapy [51]. In another study, sST2 concentration changed significantly in 37 CTEPH patients treated with BPA in the immediate postprocedural period. Interestingly, in patients who experienced complications in the postprocedural period, the baseline sST2 levels were significantly higher in comparison to those without complications. Thereby, sST2 could be beneficial for preoperative risk assessment in these patients. Furthermore, sST2 concentration significantly increased early after BPA procedure, irrespective of complications. In contrast, no analogous changes in NT-proBNP levels were noticed, which may be suggestive of an additional noncardiac source of sST2 in CTEPH patients. Therefore, in PH, sST2 as a complex biomarker may reflect not only the heart condition but also pulmonary vascular system and lung tissue [52]. Table 3 summarizes the main differences between sST2 and NT-proBNP in management of PH.

### 2.4. Heart-Type Fatty Acid-Binding Protein

Heart-type fatty acid-binding protein (H-FABP) is a low-molecular-weight protein, which is expressed in the cytosol of cardiomyocytes. H-FABP appears to be a marker of injury of cardiomyocytes and is also considered as additional biomarker for early diagnosis of acute coronary syndrome [53]. Of note, Puls et al. described H-FABP as a suitable marker for risk assessment in patients with acute pulmonary embolism [54]. However, there are only limited data about the application of H-FABP in PH patients. Lankeit et al. examined the role of H-FABP in risk stratification in CTEPH patients. The results of the study revealed H-FABP as an independent marker of adverse outcomes, defined as persistent PH after PEA, CTEPH-related death, or lung transplantation [55]. In contrast, Mirna et al. identified H-FABP as an indicator of postcapillary, but not precapillary PH [56]. Although these initial reports appear promising, further studies enrolling a larger population are needed in order to evaluate existing discrepancies.

## 3. Markers of Inflammation

There is increasing evidence that inflammation processes have great significance in the pathophysiology of PH, being involved in pulmonary arterial remodeling. However, the inflammatory component could also mirror organs distress caused by a certain degree of ischemia and elevated sympathetic drive as a consequence of limited cardiac output. A variety of both anti- and proinflammatory molecules have been investigated as potential biomarkers in cohorts of PH patients.

### 3.1. C-Reactive Protein

C-reactive protein (CRP), a widely used marker of inflammation, is broadly established as a predictor of numerous cardiovascular diseases, including different types of PH. In PAH, significant correlations between CRP and RAP, 6MWD as well as NYHA class were revealed [57]. Scognamiglio et al. observed that in patients with congenital heart disease-associated PAH (CHD-PAH), CRP concentration was commonly increased and the CRP elevation above 10 mg/mL was associated with around four times greater risk of death [58]. Wynants et al. examined CRP effects on pulmonary vascular cells in CTEPH patients. They revealed that CRP could play a role in chronic obstruction of pulmonary arteries by stimulating endothelial dysfunction, vascular remodeling, and in situ thrombosis [59]. In CTEPH patients, plasma CRP concentrations were related to tissue factor (TF) antigen, suggesting the connection between thrombosis and inflammatory processes in the pathogenesis of CTEPH [60]. Moreover, Quarck et al. observed that CRP levels were elevated among CTEPH patients and significantly decreased 12 months after PEA [57]. However, due to the reported elevated CRP levels in many clinical conditions, including various cardiovascular diseases, its potential use in the diagnosis and monitoring of PH patients remains limited.

### 3.2. Red Blood Cell Distribution Width

Red blood cell distribution width (RDW) is a laboratory biomarker of heterogeneity, regularly measured in standard blood analyses. Elevated RDW levels are the sign of anisocytosis, which is linked with underlying inflammatory processes [61]. So far it is known that RDW may be a predictor of survival in various cardiovascular diseases, such as coronary artery disease [62], chronic heart failure [63], or acute pulmonary embolism [64]. Moreover, RDW is a prognostic marker of PH of different etiologies, and an association with mortality in a cohort of PH patients was noticed [65,66]. In study involving 77 inoperable CTEPH and PAH patients, the decrease in RDW level after initiation or escalation of specific treatment was linked with good treatment response and improved prognosis [67]. Similar results were previously obtained by Wang et al. in 56 CTEPH patients [68]. However, there is a need for prospective studies to better assess the prognostic value of RDW in cohorts of patients with precapillary PH.

### 3.3. Growth Differentiation Factor-15

Growth differentiation factor-15 (GDF-15) is a member of the TGFβ superfamily. GDF-15 is exposed in various types of cells in response to tissue damage, ischemia, or shear stress [69,70]. So far, GDF-15 has been indicated as a nonspecific marker of systemic stress in several cardiovascular diseases [71]. In PH, GDF-15 is present in the plexiform lesions in the pulmonary vascular bed and may thus affect both apoptosis and proliferation of endothelial cells [69]. Nickel et al. revealed that in 22 patients with iPAH, GDF-15 levels were associated with hemodynamic parameters such as RAP and pulmonary capillary wedge pressure (PCWP), as well as with biochemical parameters, such as NT-proBNP concentration. However, there were no significant changes in median GDF-15 levels measured prior to beginning of specific therapy and at three- or six-month follow-up [72]. Furthermore, in a study by Meadows et al. in patients with scleroderma and associated PAH, GDF-15 was a marker of reduced survival and correlated with NT-proBNP levels and right ventricular systolic pressure assessed by transthoracic echocardiography [73]. The observations mentioned above brightly propose that GDF-15 could be a prognostic factor in PAH. GDF-15 was also assessed as a marker in therapy response in CTEPH patients treated with BPA. Kriechbaum et al. revealed no significant changes before and after BPA treatment, but there was a correlation between delta change in GDF-15 levels and the change in CI and RAP. In addition, a low concentration of GDF-15 measured at baseline indicated responders to the BPA therapy at the follow-up [51].

### 3.4. Cytokines

Various cytokines are considered crucial inflammatory mediators in numerous conditions, including PH. In a study conducted by Soon et al., serum levels of several interleukins (IL), such as IL-1, IL-2, IL-4, IL-6, IL-8, Il-10, and IL-12p70, and tumor necrosis factor-α (TNFα) were higher in patients with PAH in comparison to a group of healthy controls. From the ILs mentioned above, IL-6, IL-8, IL-10, and IL-12p70 were prognostic factors of poor survival in iPAH and familial PAH [74]. These data are consistent with results obtained by Selimovic et al., which revealed significantly higher levels of IL-6, transforming growth factor β1 (TGFβ1), platelet-derived growth factor (PDGF), and vascular endothelial growth factor (VEGF) in PAH patients compared to controls. Moreover, in this study, a significant association between IL-6 and mortality was observed [75]. Similar observations were previously revealed by Langer et al. in a cohort of CTEPH patients [76]. Elevated levels of IL-6, IL-8, and TNFα were observed in CTEPH patients before PEA. Hence, both IL-6 and Il-8 presented a noticeable peak immediately after PEA, whereas TNFα levels significantly decreased within 24 h after the procedure [76]. What is more, in a study conducted by Zabini et al., significant correlations of IL-6 and hemodynamic parameters and exercise capacity were observed [77]. As mentioned above, numerous cytokines have been investigated as potential biomarkers in PH patients, but their applicability remains still in the research phase.

### 3.5. Neopterin

Neopterin (NP), belonging to the class of pteridines, is a marker of cellular immune response. NP is predominantly released by dendritic cells and macrophages after stimulation with interferonγ (IFNγ) via guanosine triphosphate (GTP) cyclohydrolase I [78]. The crucial pathophysiological role of NP is presumably the interaction with reactive oxygen or nitrogen intermediates, in that way stimulating oxidative stress [79]. Elevated levels of NP have been observed in various clinical conditions, including cardiovascular diseases, such as heart failure and coronary artery disease [80,81]. Moreover, in the above-mentioned conditions, NP has been documented as a biomarker associated with death and adverse-event prognosis. NP could participate in the progression of various types of PH by intensifying effects of reactive oxygen species. However, there are only limited data about NP used as a biomarker in patients with PH. In the study including 50 PAH and inoperable CTEPH patients, NP levels were significantly higher in comparison to healthy controls. Moreover, positive correlations of NP with NT-proBNP, right atrium area, and negative correlations with 6MWD and peak-VO2 assessed in cardiopulmonary exercise test (CPX) were reported. Additionally, elevated NP levels were associated with poor clinical outcomes in a cohort of PH patients [82].

### 3.6. Galectin 3

Galectin-3 (Gal-3) is a beta-galactoside-binding lectin, expressed in the inflammatory cells (macrophages, neutrophils, eosinophils, and mast cells) and endothelial cells in response to tissue damage. Gal-3 is considered to be mediator of inflammatory processes and fibrosis, and its activity results in increased adverse cardiac remodeling [83,84]. Healthy cardiomyocytes are characterized by low Gal-3 expression, whereas in patients diagnosed with heart diseases, Gal-3 levels increase with disease severity [85]. In recent years, many studies have aimed to demonstrate the role of Gal-3 in diagnosis and prognosis of left-sided HF. Multiple measurements of Gal-3 levels in two large cohorts of patients with chronic and acute HF provided important information on the prognostic value of Gal-3 in identifying high risk of morbidity and mortality [86,87,88]. However, Gal-3 may be suitable also for monitoring right ventricular remodeling [89]. There are only limited data about the role of Gal-3 measurements among PH patients, focusing mainly on patients with PAH and consisting of small cohorts. Calvier et al. demonstrated high levels of Gal-3 in PAH patients and revealed correlations between Gal-3 and functional parameters [90]. Mazurek et al. observed that elevated levels of Gal-3 were associated with mortality in PH patients, but the study involved both PAH and PH due to left-sided HF patients [91]. In the study conducted by Geenen et al., including 164 patients with PAH, CTEPH, or PH caused by lung disease, there were no significant differences in the Gal-3 levels between subgroups [92]. However, higher levels of Gal-3 were associated with adverse outcomes, defined as death, hospitalization, or lung transplantation. In another study, Gal-3 was evaluated in relation to disease severity and treatment response in patients with CTEPH treated with BPA. In the context of risk assessment and evaluation of therapy response, no diagnostic benefits were revealed [51].

## 4. Biomarkers Related to Pulmonary Arterial Smooth Muscle Cell and Endothelial Dysfunction

Although the pathogenesis of PAH is fundamentally different from that of CTEPH, it has been shown that changes in pulmonary arterial microcirculation can be similar in both types, including excessive pulmonary arterial smooth muscle cell (PASMC) proliferation and endothelial dysfunction. There are several biomarkers linked to endothelial cells and PASMC studied in the PH patient population.

### 4.1. Asymmetric Dimethylarginine

Asymmetric dimethylarginine (ADMA), produced by endothelial cells, is a competitive nitric oxide synthase inhibitor [93]. As we know from several reports, ADMA levels are higher and are associated with adverse outcomes in patients with different types of PH, including idiopathic PAH and CTEPH, but also with other diseases, including acute myocardial infarction [94]. In study conducted by Kilestein et al., ADMA correlated with CI, RAP, and mvSatO2 and was an independent predictive factor of mortality in PAH [95]. Similar correlations were achieved by Skoro-Sajer et al. in a cohort of CTEPH patients [96]. Furthermore, Sanli et al. revealed higher ADMA concentrations in pediatric patients diagnosed with congenital heart disease [97].

### 4.2. Endothelin-I and COOH-Terminal Pro Endothelin 1

Endothelin-I (ET-I), involved in cardiovascular hemostasis and respiratory development, promotes PASMC proliferation and migration and is considered an effective vasoconstrictor [98,99,100]. In patients with different types of PH, high levels of ET-1 and significant correlations of ET-1 with hemodynamic parameters are observed. At present, in the treatment of PAH, endothelin receptor antagonists are well established and commonly used with favorable effects [98,101]. In CTEPH, preoperative high ET-1 levels correlate positively with clinical severity of the disease, being potential predictors of hemodynamic effects after PEA [102]. These observations were also confirmed by Reesink et al., whose study showed ET-1 levels in CTEPH patients were useful for identifying patients at risk for residual or persistent PH after surgical treatment [103]. However, there are some specific obstacles that make ET-1 not an attractive biomarker. These obstacles can be overcome by COOH-terminal pro endothelin 1 (CT-proET-1). Firstly, there is no correlation between ET-1 levels in lung and plasma, which may be explained by its paracrine functions [104]. Secondly, direct measurement of ET-1 levels can be affected by cross-reactivity. On the contrary, CT-pro-ET-1 is present in plasma and remains stable at room temperature for a few hours [105]. In the study performed by Marques et al., including 28 patients with PAH, plasma CT-pro-ET-1 levels were associated with functional parameters and were predictors of hospitalization, death, or lung transplantation within 12-month follow-up [106].

### 4.3. MicroRNAs as Biomarkers in PAH

MicroRNAs(miRNAs) are small, endogenously expressed noncoding RNAs, circulating in the blood. MiRNAs regulate gene expression at the posttranscriptional level by degrading or stopping target RNAs [107]. MiRNAs measurements may play a role as biomarkers for several pathologies, including HF or acute myocardial infarction [108,109]. Finally, miRNAs are engaged in the progression of iPAH, including plexiform lesion creation, endothelial dysfunction, smooth muscle cell proliferation, and both activation and proliferation of fibroblasts [110]. There is evidence for miRNAs as markers of PAH progression. Rothman et al. revealed reduced levels of miR-140-5p in PAH patients in comparison to control group. Further, inhibition of miR-140-5p promotes PASMC proliferation and migration in vitro, which may be relevant in the progression of the disease [111]. Sarrion et al. assessed circulating miRNAs in 12 patients with idiopathic PAH. As a result, they found significant changes in 61 miRNAs. Additionally, correlations between the expression of miR23a and hemodynamic parameters, such as mean PAP, CI, and PVR, were observed. Additionally, the expression profile of circulating messenger RNA (mRNAs) was studied, and results revealed that miR23a controlled 17% of the significantly changed mRNA, including PGC1𝛼, which is related to the progression of iPAH [112].

## 5. Markers of Hypoxia and Tissue Damage

The natural course of PH leads to low cardiac output and respiratory mismatch, which in sequence turn to peripheral hypoperfusion. Thereby, biomarkers mirroring peripheral damage and hypoperfusion, such as partial pressure of carbon dioxide (pCO2) and copeptin, could be tools in monitoring disease progression and ascertaining prognosis.

### 5.1. pCO2

As is well known, pCO2 levels are constantly decreased in patients with different types of PH. In accordance to a retrospective study conducted by Hoeper et al., decreased pCO2 levels in PAH patients were associated with poor prognosis, whereas decreased pO2 levels had no prognostic significance. Interestingly, in the same study, the improvement of pCO2 after 3 months of PAH targeted therapy was noted, and that was associated with higher survival rate [113]. However, a recent study conducted by Valentin et al. revealed that in PAH patients, a ≥3% decrease in arterial oxyhemoglobin saturation (SaO2), noticed after 3 months of treatment with PAH-specific drugs, was associated with worse outcomes [114].

End-tidal partial pressure of CO2 (P_ET_CO2) is a measurement made at the airway during CPX. In normal individuals, P_ET_CO2 increases from rest to the anaerobic threshold (AT), then stabilizes during the isocapnic buffering period and finally decreases later during exercise [115]. In both PAH and CTEPH, P_ET_CO2 is frequently low at rest and declines further during early exercise. In detail, P_ET_CO2 at AT less than 30 mmHg may be suggestive for pulmonary vascular disease in patients with unexplained dyspnea, whereas values greater than 38 mmHg make PH diagnosis unlikely. Low values of P_ET_CO2 are associated with altered chemosensitivity, ventilatory inefficiency, and high dead space ventilation, due to reduced perfusion of highly ventilated alveoli. The magnitude of P_ET_CO2 reduction inversely correlated with the degree of mPAP elevation in a cohort of PH patients [116].

### 5.2. Uric Acid

Uric acid (UA) is the end product of purine metabolism and a marker of impaired oxidative metabolism. UA can be produced due to various conditions, such as cardiac overproduction, renal impairment, or use of diuretics in right-sided HF. Serum UA levels have been presented to be elevated in patients with both left- and right-sided HF [117]. Thus, the use of UA as a marker in PH remains debatable. Nagaya et al. revealed significantly increased serum levels of UA in PH patients. Further, serum UA levels were independently related to mortality [118]. In another study by Voelkel et al., patients with severe PH had higher levels of UA and UA levels were positively correlated with RAP [119]. Wang et al. assessed UA levels in a cohort of 50 patients diagnosed with PAH related to connective tissue disease (CTD-PAH). They observed that patients with increased levels of UA at baseline had a lower survival rate in comparison to those with normouricemia. It is suggestive for UA levels as a prognostic factor in CTD-PAH patients [120]. Interestingly, in pediatric PAH patients, treatment with prostacyclin was able to reduce UA levels [121]. However, it remains unclear whether treatment of increased UA concentrations has an influence on prognosis. Finally, the main limitation of UA as a marker is lack of specificity for PH.

### 5.3. Copeptin

Copeptin, the C-terminal fragment of the vasopressin’s precursor, is an emerging surrogate target for assessment of vasopressin levels. Copeptin has arisen as a valuable tool in monitoring cardiovascular pathologies. As known from previous reports, copeptin can deliver additional information to troponin in the initial assessment process of patients with chest pain [122,123,124]. In patients diagnosed with chronic HF, copeptin increases the diagnostic value of NT-proBNP as a predictor of all-cause mortality [122,125]. In PAH patients, copeptin significantly correlates with 6MWD and NYHA class as well as with kidney function [126,127,128]. Moreover, in a study performed by Nickel et al., elevated copeptin concentration was related to a higher risk of death and was an independent predictive factor of adverse outcomes in PAH patients. Noteworthy, copeptin levels were independent of hemodynamic parameters, but correlated well with NT-proBNP concentration, both at baseline and after initiation of treatment targeting pulmonary arterioles. Consequently, copeptin might mirror neurohumoral activation due to altered function of the RV. Thus, elevated copeptin levels could add significant prognostic information that is not captured by RHC or NT-proBNP concentration assessed alone [129]. Summarizing, copeptin could be an additional biomarker in multimarker testing strategies for better risk stratification in PH patients.

## 6. Extracellular Vesicles

There is increasing evidence suggesting an active role for extracellular vesicles (EVs) in the pathophysiology of PH. Extracellular vesicles have different origins, such as endothelial cells, leukocytes, and platelets [130,131]. The concentration of EVs increases differently and specifically in various pathological conditions, including cardiovascular diseases [132]. In detail, in studies conducted by Amabile et al., circulating EVs of leukocyte (CD11b+), platelet (CD31+CD61+), as well as endothelial (CD62e+) origin, measured by flow cytometry, were higher in patients with PH in comparison to controls. In addition, endothelial EVs levels correlated with the hemodynamic severity of PAH and were associated with adverse clinical events [131,133]. Moreover, PAH patients had higher endothelial EV concentrations compared to patients with chronic pulmonary disease-related PH [131]. These observations indicate the potential role of EVs in the disease onset and progression of PAH. In detail, both leukocyte and platelet EVs promote destruction of the pulmonary endothelium and remodeling of the PASMC. On the other hand, Diehl et al. reported higher levels of endothelial EVs bearing E-selectin in thromboembolic PH in comparison to non-thromboembolic PH patients [130]. Gąsecka et al. investigated EVs levels in 42 PAH patients treated with prostacyclin analogues. Patients during treatment with prostacyclin analogues had similar concentrations of EVs from platelets, but lower concentrations of EVs from leukocytes and activated platelets in comparison to patients treated with phosphodiesterase type 5 inhibitor and/or endothelin receptors antagonists. Moreover, the authors noticed a trend toward a lower concentration of EVs from endothelial cells in patients treated with prostacyclin analogues [134]. These results may suggest that the decrease of EVs levels might be one of the mechanisms underlying the favorable effects of prostacyclin analogues in PAH patients.

## 7. Other Biomarkers

### 7.1. High-Density Lipoprotein Cholesterol

High-density lipoprotein cholesterol (HDL-C) is a well-known biomarker of various cardiovascular diseases. In two studies among PAH patients, decreased levels of HDL-C were measured. Moreover, levels of HDL-C were strongly associated with survival in patients with PAH [135,136]. In a retrospective study involving 90 patients suffering from CTEPH, high levels of HDL-C significantly correlated with a decrease in right ventricular dilatation and a considerable reduction in PVR after surgical treatment [137].

### 7.2. Adiponectin

Adiponectin (APN) is an adipokine, completely produced and secreted in the adipose tissue. However, in addition to adipocytes, APN is exposed also in various other cell types, such as cardiomyocytes and endothelial cells [138,139]. In PH, APN is considered to exert a cardioprotective effect, by reducing inflammatory processes and inhibiting vascular smooth muscle cells proliferation [119]. In a study of 30 CTEPH patients, APN levels were elevated and positively correlated with hemodynamic parameters, such as PVR, and with BNP concentration. Moreover, levels of APN decreased significantly after improvement of RV function due to BPA or PEA. Thus, changes in APN levels in association with hemodynamic severity and clinical outcome are suggestive for the role of APN as a prognostic biomarker in PH [140]. However, given that the exact role of adiponectin in PH has not yet been fully understood, further studies are needed to better evaluate this biomarker.

### 7.3. F(2)-Isoprostane

F(2)-isoprostane is a marker of lipid peroxidation, measured both in urine and in plasma samples. It is regarded as an oxidative stress marker; however, its source has not been identified yet. Data assessing F(2)-isoprostane among PH patients are limited. Cracowski et al. prospectively assessed urine F(2)-isoprostane levels in 110 PAH patients. They revealed that F(2)-isoprostane was an independent predictive factor of mortality in this cohort. Moreover, they also observed that F(2)-isoprostane measurement in children with family history of PAH can help in early detection of the disease [123,141]. In another study, Zhang et al. determined F(2)-isoprostane plasma levels in 80 patients diagnosed with iPAH at the time of their first RHC and monitored them for 30 ± 12 months. F(2)-isoprostane were elevated in the study population and significant correlations with WHO functional class, 6MWD, mvSatO2, mRAP, and BNP were noticed. Moreover, F(2)-isoprostane concentration increased further in nonsurviving patients despite similar targeted treatment being administered in both survivors and nonsurvivors. Histological studies presented that the expression of F(2)-isoprostane was upregulated in remodeled pulmonary vessels in autopsy lung samples [142].

## 8. Conclusions

The present article reviews several promising biomarkers that have been identified and assessed in PH patients over the past few years. Although a wide range of biomarkers have been explored, none of them are specific enough to be used alone in the diagnostic process and prognosis assessment. Moreover, the majority of biomarkers mentioned in this review have been evaluated in retrospective studies with a small number of patients and/or controls, divergent studied population, and with potential selection bias. Therefore, part of the available data should be taken with caution. Larger, prospective clinical trials are needed to validate potential biomarkers for both diagnosis and prognosis. Furthermore, given the complexity of the disease, it remains unlikely that a single biomarker can be used successfully in patients with precapillary PH. Multimarker strategies for risk assessment, as applied in patients with left HF, should be investigated and validated in PH.

## Figures and Tables

**Table 1:** Changes in BNP levels in CTEPH patients before and after BPA treatment in the hitherto published case series.

Studies	No. of Patients (n)	No. of BPA Sessions (n)	BNP before BPA (pg/mL)	BNP after BPA (pg/mL)	*p*
Sugimura et al. [18]	12	NR	335 ± 105	16 ± 11	S
Kimura et al. [19]	66	446	237.7 ± 475.7	45.2 ± 47.6	S
Ogo et al. [20]	80	385	227 ± 282	48 ± 57	S
Yamasaki et al. [21]	20	2.7 per pt	66.5 ± 61.3	33.8 ± 30.0	S
Aoki et al. [22]	24	113	112 (49–199)	27.5 (14.6–58.4)	S
Inami et al. [23]	103	350	94 (42–232)	61 (39–150)	S

Data are presented as mean ± SD or median and (IQR), S—*p* < 0.05; BPA—balloon pulmonary angioplasty; BNP—brain natriuretic peptide.

**Table 2:** Changes in NT-proBNP levels in CTEPH patients before and after BPA treatment in the hitherto published case series.

Studies	No. of Patients (*n*)	No. of BPA Sessions (*n*)	NT-proBNP before BPA (pg/mL)	NT-proBNP after BPA (pg/mL)	*p*
Kurzyna et al. [24]	31	117	2571 ± 2719	634 ± 697	S
Olsson et al. [25]	66	446	504 (233–1676)	242 (109–555)	S
Araszkiewicz et al. [26]	15	71	1554.8 ± 1541.3	537 ± 642.6	S
Darocha et al. [27]	70	377	1307 (510–3294)	206 (83–531)	S
Gerges et al. [28]	45	6 (4–10) per pt	579 (182–1385)	198 (70–429)	S

Data are presented as mean ± SD or median and (IQR), S—*p* < 0.05; BPA—balloon pulmonary angioplasty; NT-proBNP—N-terminal-pro brain-type natriuretic peptide.

**Table 3:** Main differences between NT-proBNP and soluble ST2 as biomarkers in PH.

Feature	Soluble ST2 Protein	BNP/NT-proBNP
Origin	The Toll-like receptor superfamily binding IL-1; Il33–ST2 pathway	Oligopeptide nuerohormones
Source of secretion	Cardiomyocytes, endothelial cells, inflammatory cells	Cardiomyocytes
Form	Two isoforms: transmembrane ST2-ligand (ST2L) and soluble ST2 (sST2)	N-terminal fragment of prohormone
Physiological function	Cardioprotective role, enhancement of Th2-dependent immune response	Cardiovascular homeostasis, vasodilatation
Pathophysiological basis	Cardiac remodeling and fibrosis	Hemodynamic condition
Secretion factor	Hemodynamic stress and myocardial remodeling; inflammation	Pressure and volume overload
Age- and renal function-dependence	NO	YES
Role in diagnosis of PH	NO	YES
Role in prognosis of PH	YES	YES
Correlation with disease severity	YES	YES
Correlation with treatment effect	YES	YES

BNP—brain natriuretic peptide; NT-proBNP—N-terminal-pro brain-type natriuretic peptide.

## Data Availability

All of the cited data in this review has come from professional medical literature cited in the Reference section according to order of appearance. Data that describe the experience of this review’s authors have also been properly cited, as they come from our peer-reviewed published articles and book chapters.
